# Status of antimicrobial resistance in food animals in Pakistan (2016–2020): A systematic review and meta-analysis

**DOI:** 10.5455/javar.2025.l930

**Published:** 2025-06-02

**Authors:** Muhammad Javed Arshed, Muhammad Umair, Usman Talib, Muhammad Farooq Tahir, Muhammad Abubakar, Sami Ullah Khan Bahadur, Tahmeena Tahmeena, Riasat Wasee Ullah, Mashkoor Mohsin, Muhammad Athar Abbas, Qadeer Ahsan, Javaria Alam, Muhammad Usman Zaheer

**Affiliations:** 1 National Veterinary Laboratory, Ministry of National Food Security and Research, Islamabad, Pakistan; 2 Institute for Antimicrobial Research, Department of Biology, University of Oxford, Oxford, UK; 3 Fleming Fund Country Grant Pakistan, Islamabad, Pakistan; 4 Integral Global, Atlanta, GA; 5 College of Veterinary Medicine and Biomedical Sciences, Colorado State University, Fort Collins, USA; 6 Dopasi Foundation, Islamabad, Pakistan; 7 Livestock Wing, Ministry of National Food Security and Research, Islamabad, Pakistan; 8 Institute of Microbiology, University of Agriculture, Faisalabad, Pakistan; 9 National Reference Laboratory for Poultry Diseases, National Agricultural Research Centre, Islamabad, Pakistan

**Keywords:** Multidrug-resistant bacteria, systematic review, meta-analysis, food animals Pakistan, *Escherichia coli*, *Salmonella*

## Abstract

Antimicrobial resistance (AMR) is a global public health issue, causing an estimated 1.27 million deaths in 2019. This systematic review and meta-analysis aim to assess the burden of AMR in food animals in Pakistan, identify resistant microbes, and highlight emerging trends in multidrug resistance (MDR). The major databases were searched for articles published between 2016 and 2020 on the prevalence of AMR in food animals in Pakistan. A random-effects model was employed to pool the prevalence of antibiotic-resistant Enterobacteriaceae and non-Enterobacteriaceae pathogens. Among 1,145 studies, 35 met the inclusion criteria as evidence of AMR in food animals. *Escherichia coli* showed the highest resistance to ampicillin (59.5%), ciprofloxacin (49%), oxytetracycline (39%), and chloramphenicol (35%); *Salmonella* to ampicillin (78.4%), amoxicillin (53.9%), chloramphenicol (40%), tetracycline (39.3%), and ciprofloxacin (39%); *Staphylococci* to cefoxitin (53.8%) and penicillin (34.8%); and *Campylobacter* and *Klebsiella* to ciprofloxacin (50.4% and 83.3%, respectively). MDR was observed in *E*. *coli* (12/12 studies), *Salmonella* (7/10), *Staphylococci* (3/8), *Campylobacter* (3/3), and *Klebsiella* (1/3), with extensive drug resistance in *E. coli* (3/12), *Salmonella* (4/10), *Campylobacter* (1/3), and *Klebsiella* (2/2). Enterobacteriaceae showed significant resistance to tetracyclines (pooled prevalence/PPr = 0.75) and aminopenicillins (PPr = 0.74), whereas non-Enterobacteriaceae showed resistance to cephalosporins (PPr = 0.67) and aminopenicillins (PPr = 0.59), both with substantial heterogeneity. This review shows the existence of bacteria resistant to commonly used antimicrobials in food animals, potentially a threat to both human and animal health. The findings suggest the continuous monitoring of AMR and antimicrobial use (AMU) and the regulation of AMU in the food and agriculture sectors.

## Introduction

Antimicrobial resistance (AMR) is an ever-growing global health challenge that has been brought to the international agenda, reinforcing collaboration across countries and sectors (human, animal, plant, and environmental) to strategize effective and sustainable mitigation plans [1]. AMR threatens public health, socioeconomic development, and environmental sustainability [2]. The emergence of AMR is attributed to selective pressure from the extensive use of antimicrobials in various domains, such as communities, hospitals, veterinary health, agriculture, aquaculture, and the environment [3]. This situation is further aggravated by the low investment in developing new antibiotics [4]. Animals are widely recognized as major reservoirs of antibiotic-resistant bacteria [5] due to the extensive utilization of antibiotics for therapeutic, preventive, and growth-promoting purposes. This practice ultimately contributes to the emergence, selection, and dissemination of antimicrobial-resistant microorganisms in animals. Consequently, there is a potential risk of transmission to humans through zoonotic infections or the food chain [6,7]. The presence of AMR in food animals threatens public health and food safety. It jeopardizes attaining several United Nations’ Sustainable Development Goals, contextualizing AMR as a global threat [8]. Approximately 60% of pathogens are shared between humans and animals through the environment [9].

Similarly, pet birds are also potential reservoirs for zoonotic transmission of pathogens within the One Health framework [10]. To mitigate the escalation of AMR, the Food and Agriculture Organization of the United Nations (FAO), the World Organization for Animal Health (WOAH), the World Health Organization (WHO), and the United Nations Environment Programme collectively advocate for the implementation of the One Health approach, as outlined in key policy documents. These include the WHO Global Action Plan on AMR [11], the OIE Strategy on AMR and Prudent Use of Antimicrobials [12], and the FAO Action Plan on AMR [13]. These endorsed frameworks provide guidance and strategies to comprehensively address AMR across human health, animal health, and the environment.

Over the past several years in Pakistan, AMR and associated challenges have recently been highlighted, with the evidence showing extensive misuse of antimicrobials in public and private sectors [14]. Furthermore, the emergence of multidrug-resistant (MDR) microorganisms, often referred to as “superbugs,” can be influenced by practices outside the formal healthcare sector, including those of unlicensed practitioners. Hence, several actions have been taken by the Government of Pakistan, including the development of the National Action Plan (NAP) [15], the National Strategic Framework for Containment of AMR [16], and the establishment of AMR surveillance networks in human and animal health sectors. The Ministry of National Health Services Regulations and Coordination, the Ministry of National Food Security and Research, the Ministry of Climate Change, the Provincial and Regional Health and Livestock Department, the Drug Regulatory Authority of Pakistan, academia, development partners, and other stakeholders from the public and private sectors are major stakeholders in Pakistan in implementing the NAP for AMR [15].

Although Pakistan’s government has committed to the NAP for addressing AMR, substantial information gaps regarding AMR magnitude, surveillance, and transmission chains limit the effectiveness of these strategies and efforts [17]. A deeper understanding of the spillover, diversity, and complex factors associated with AMR at the national level is required to drive the agenda ahead. Presently, most data on AMR come from the developed world, and efforts in low- and middle-income countries should be encouraged where the AMR burden is very high [18]. Such an effort requires national and international collaborative approaches, which can lead to an in-depth assessment of the problem burden and design of intersectoral interventions to achieve the five objectives of the WHO-2015 Global Action Plan [11].

Various studies on AMR have been conducted in Pakistan by different academic and research institutions and organizations that share the findings in national and international journals. However, the dynamics of AMR at the human–animal–environment interface remain unclear because of a lack of consolidated data and well-defined and case-controlled studies. This hampers attempts to build and operationalize comprehensive strategies for the country. To consolidate country AMR data, we conducted this systematic review and meta-analysis aimed at understanding the landscape of resistant bacteria and associated antimicrobials to assess the burden of AMR in food animals and to analyze the resistance patterns of antibiotic classes across bacterial species to understand their practical use and applicability. This review contributes to the way forward for developing key recommendations, future policy decisions, and specific action plans and interventions. By synthesizing available data, this study aims to bridge existing knowledge gaps and support evidence-based strategies for AMR containment in Pakistan.

## Materials and Methods

The review was prepared according to the guidelines provided by the Preferred Reporting Items for Systematic Reviews and Meta-Analyses [19].

### Search strategy

Four databases—Google Scholar (GS), PubMed, Web of Science (WoS), and CAB—were searched on December 17, 2020, to retrieve relevant studies published from 2016 to 2020 in Pakistan. PubMed focuses on biomedical and clinical research, Web of Science covers multidisciplinary studies, and CAB specializes in agriculture and veterinary science, ensuring comprehensive coverage of both medical and agricultural literature. The timeframe was selected because it coincided with key global and national AMR policy developments, following the World Health Assembly’s adoption of the Global Action Plan on AMR in 2015. Therefore, this period marked the onset of substantial AMR surveillance and research in Pakistan, establishing a baseline for assessing the AMR burden in food animals and providing a foundation for evaluating the impact of later policy-driven interventions. The search strategy used in all databases was a combination of the terms [antimicrobial resistant* OR antibiotic resistant* OR multidrug resistant*] AND (food animal* OR farm animal* OR domestic animal* OR bovine OR livestock OR cattle OR buffalo* OR sheep OR goat OR broiler OR layer OR poultry) AND Pakistan. We selected these key terms to ensure thorough coverage of relevant publications. The search results were organized and managed using Microsoft Excel (2016).

### Eligibility criteria

All studies included in the review, which were conducted in Pakistan, assessed AMR in food animals (domestic animals, farm animals, cattle, buffaloes, sheep, goats, and poultry). The review focused on studies reporting the phenotypic resistance of bacteria in food animals and/or related markets/products (such as milk, meat, and eggs). Resistance to β-lactams, aminoglycosides, and flu-oroquinolones was of particular concern, and antibiotics were classified as critically important for both human and animal health according to the WHO and the WOAH [20]. MDR bacteria and antimicrobial-resistant gene detection mechanisms were determined. Records that did not meet the inclusion criteria, such as studies focused on parasites, viruses, and fungi, as well as those investigating AMR in aquatic animals, companion animals, wildlife, and the environment, were excluded. Studies reporting data from outside Pakistan, published before 2016, unpublished data, reviews, posters, abstracts, letters to editors, book chapters, and reviews were also excluded.

### Screening criteria

A four-step process was used to filter the search results. In step 1, all duplicate records among and within the data-bases were identified and removed. In step 2, records were screened, and those in the non-English language were removed. In step 3, records were assessed for originality; only research-reported primary data published in peer-reviewed journals were retained, and all other irrelevant records were removed. Step 4 was performed by two independent reviewers who screened the abstracts and titles, followed by a full-text review of the relevant studies based on the eligibility criteria. Any disagreement between the reviewers who independently assessed the records was resolved through discussion. To ensure the quality of the studies included, only peer-reviewed articles with primary data published in reputable journals were considered. However, relying on publicly available datasets may introduce limitations, such as variability in study design, reporting practices, and data collection methods.

### Framework for data extraction

Data were extracted and analyzed using a standardized framework in Microsoft Office Excel 2016. The collected information included details such as author information, study location (province/region), publication year, research objectives, study population (e.g., poultry, cattle, sheep, and goats), sample types (e.g., postmortem specimens, poultry organs, cloacal/rectal/nasal swabs, fecal samples, milk, eggs, raw meat), sample size, clinical status (e.g., study design (single or multisite), target bacteria (e.g., *Salmonella* spp., *Escherichia coli*, *Staphylococcus aureus*, *Campylobacter*, *Klebsiella*), tested antibiotics, antimicrobial susceptibility testing (AST) methods (disk diffusion, micro-broth dilution, agar dilution, E-test), interpretation guidelines for AST [e.g., EUCAST, Clinical and Laboratory Standards Institute (CLSI), National Committee for Clinical Laboratory Standards (NCCLS)], and the prevalence of AMR/MDR and corresponding results. We contacted the authors to clarify the study’s findings if needed. Articles that evaluated numerous outcomes were limited to those relevant to the focus of this review.

### Meta-analysis

Meta-analysis was performed to assess the prevalence of AMR in Enterobacteriaceae and non-Enterobacteriaceae using R [21] with the “metafor” package in RStudio. *E. coli, Salmonella*, and *Klebsiella* were categorized as subgroups under Enterobacteriaceae, whereas *Staphylococcus* and *Campylobacter* were categorized as non-Enterobacteriaceae. The effect size for each study was calculated using the argument “IR” for the raw incidence rate. IR is the proportion of positive outcomes among the total number of tests, and the IR in this meta-analysis represents the proportion of resistant isolates among the isolates tested in each study. The pooled IR for each group and subgroup was calculated as the total number of positive isolates among the isolates tested in all studies. IR and pooled IR correspond to the prevalence (Pr) and pooled prevalence (PPr) of different studies and groups or subgroups in this meta-analysis. The level of heterogeneity among studies in each group and subgroup was presented using the tau-square (*τ^2^*), Cochran’s Qm, and *I^2^* statistics. *τ^2^* represents the variance in the effect size among studies and quantifies how much the effect sizes of individual studies differ from the overall mean; Q tests whether the variation across studies is greater than what would be expected by chance, and high Q indicates greater variability among studies; and *I^2^* measures the percentage of total variation across studies due to heterogeneity rather than chance. Cochran’s statistic for subgroup heterogeneity, denoted as Qm, represents the weighted sum of the squared deviations of the subgroup mean from the overall grand mean. To evaluate publication bias, funnel plots were used, which visually assess whether smaller studies are systematically missing, often due to the lack of publication of negative or inconclusive results. Run plots of influence diagnostics were created using the same metafor package to identify those influencing RE meta-analysis results. Due to the nature of the included studies, which were not randomized trials, and the outcome being based on standardized culture tests, GRDAE analysis was not performed.

## Results

**Figure 1.**
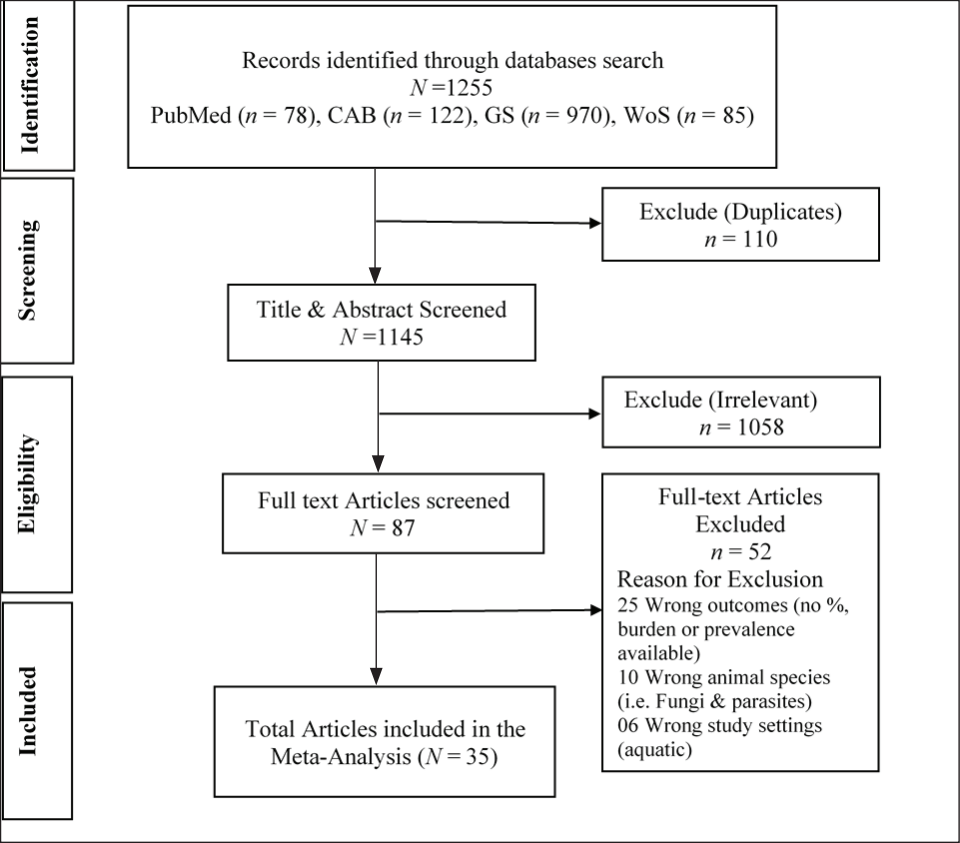
PRISMA flow diagram of the literature search, screening, and inclusion/exclusion criteria.

The database search yielded 1,255 studies from Google Scholar (*n =* 970), CAB (*n =* 122), PubMed (*n =* 78), and Web of Science (*n =* 85). A total of 110 duplicate studies were identified across multiple databases (GS: *n =* 8, CAB: *n =* 27, PubMed: *n =* 13, and WoS: *n =* 52). Before screening, a calibration exercise was conducted to ensure consistency among the reviewers in applying the inclusion and exclusion criteria. After eliminating duplicate records, 1,145 studies were screened based on their language and originality in research, and 1,058 studies did not meet the inclusion criteria during the title or abstract screening process. A total of 87 studies that met the inclusion criteria were subjected to a full-text review. Of these, 36 studies that did not report AMR outcomes in animals were excluded from further analysis. During the data extraction phase, an additional 16 studies were excluded because of outcomes reported for parasites, fungi, and aquatic animals. The 35 remaining records were included in this meta-analysis (Fig. 1).

### AMR landscape

The 35 included studies presented a diverse provincial distribution, with studies reported in Punjab (*n =* 19), Khyber Pakhtunkhwa (*n =* 11), Sindh (*n =* 2), Baluchistan (*n =* 1), Islamabad (*n =* 1), and multiple regions (*n =* 1). The sites of the included studies that reported AMR were poultry farms (*n =* 10), poultry markets (*n =* 11), and livestock (*n* = 5). One study reported on AMR in camels. The remaining eight studies reported AMR from multiple sites, including livestock and poultry farms, as well as from the environment. Poultry specimens (liver, kidney, intestinal contents, cloacal swabs, and droppings), eggs, meat, milk, and feces were included in the included studies.

The most common isolate was *E*. *coli* (12 studies and a total of 3,054 samples), with 1,161 (38.0%) antibiotic-resistant cases. For *Salmonella* studies (*n =* 10), 2,630 samples were analyzed, revealing 815 (31.0%) resistance-positive cases. In the case of *Staphylococci* (*n =* 8), which examined 3,121 samples, of which 719 (23.0%) resistance-positive samples were reported. For *Campylobacter* (*n =* 3) studies, a total of 2,060 samples were reported, yielding 387 (19.0%) resistance-positive cases. Finally, *Klebsiella* studies (*n =* 2) encompassed 338 samples, of which 60 were reported positive (18%) (Table 1). The isolates were tested for the presence of different classes of antibiotics. The most common method used to assess antibiotic resistance was the disk diffusion method, employed in 34 studies, and microdilution in one study. Resistance breakpoints were identified according to the CLSI in 32 studies, the NCCLS in two studies, and EUCAST in one study.

**Table 1.** Prevalence of antibiotic resistance in different bacterial isolates among food animals: A summary of study findings.

Pathogen	Number of studies	Sample size	Samples positive number (%)
*E. coli*	12	3054	1161 (38%)
*Salmonella*	10	2630	815 (31%)
*Staphylococci*	08	3121	719 (23%)
*Campylobacter*	03	2060	387 (19%)
*Klebsiella*	02	338	60 (18%)

### AMR pattern

In the 35 included studies, 46 antibiotics were assessed for resistance against *Staphylococci* and *Salmonella*, while 22 antibiotics were evaluated against *Campylobacter* and *Klebsiella* spp. Among the *Staphylococci* studies, 41 antibiotics were tested, while 22 antibiotics were tested against *Campylobacter* and *Klebsiella* spp. isolates.

Among the total antibiotic tests for *E*. *coli*, the highest percentage of positive resistance was reported for ampicillin (59.5%), followed by ciprofloxacin (49.0%), oxytetracycline (39.0%), and chloramphenicol (35.0%). For *Salmonella*, positive resistance was found highest for ampicillin (78.4%), followed by amoxicillin (53.9%), chloramphenicol (40.0%), tetracycline (39.3%), and ciprofloxacin (39.0%). In the case of *Staphylococci*, the highest resistance was reported against cefoxitin (53.8%), followed by penicillin (34.8%) and oxytetracycline (27.1%). The *Campylobacter* and *Klebsiella* isolates exhibited high resistance to ciprofloxacin, with resistance rates of 50.4% and 83.3%, respectively.

### MDR patterns in food animals

Studies reporting MDR revealed that *E*. *coli* was resistant to multiple drugs in all 12/12 studies, *Salmonella* in 7/10 studies, *Staphylococci* in 3/8, *Campylobacter* in 3/3, and *Klebsiella* in only 1/2 studies. Extensive drug resistance (XDR) was reported in *E*. *coli* 3/12, *Salmonella* 4/10, *Campylobacter* 1/3, and *Klebsiella* 2/2. More sophisticated analyses were conducted to identify the resistance genes for *E*. *coli* in 6/12 studies, *Salmonella* in 3/10 studies, *Campylobacter* in 2/3 studies, and *Klebsiella* in 1/2 studies.

### Meta-analysis

#### Phenotypic resistance among Enterobacteriaceae

**Figure 2.**
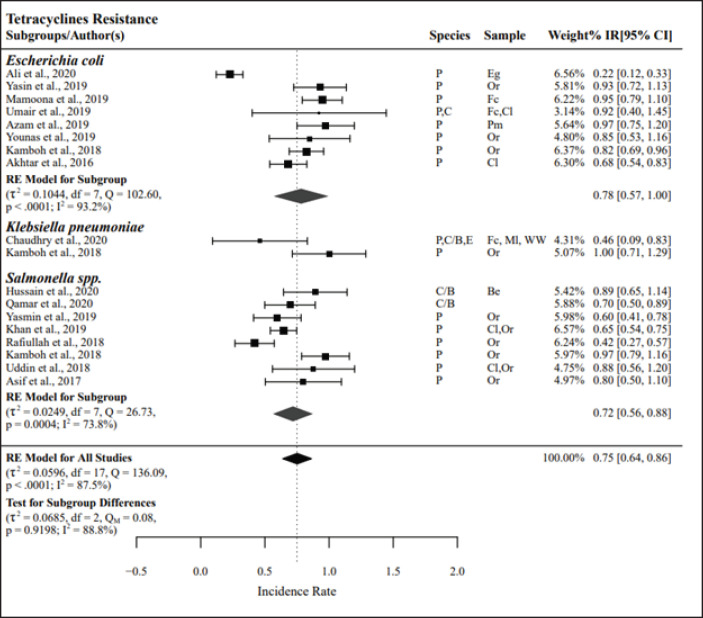
Pooled prevalence rates of antibiotic resistance for *Enterobacteriaceae.* IR, raw incidence rate; *I*^2^ = heterogeneity; RE, random effects model; Q, heterogeneity statistics.

**Figure 3.**
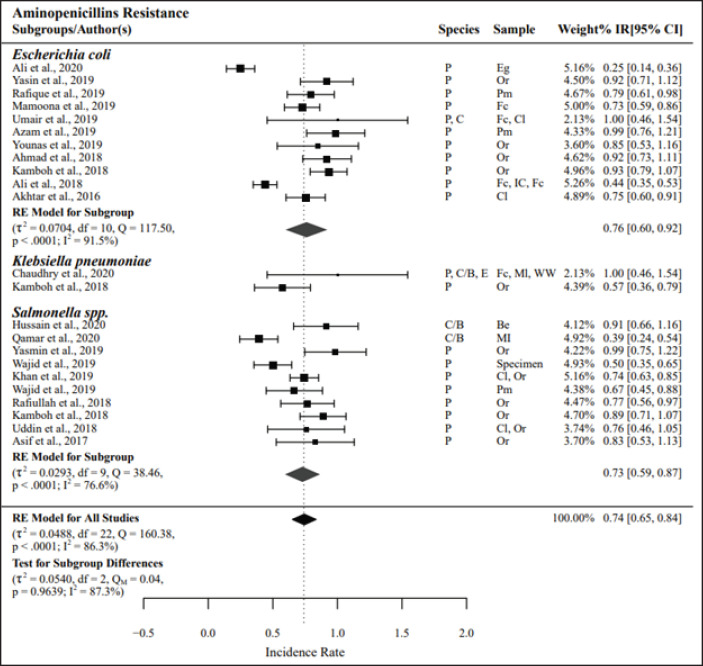
Pooled prevalence rates of antibiotic resistance for *Enterobacteriaceae.* IR, raw incidence rate; *I*^2^ = heterogeneity; RE, random effects model; Q, heterogeneity statistics.

The meta-analysis results for Enterobacteriaceae groups tested against tetracyclines (*n =* 18) and aminopenicillins (*n =* 23) were found to have the highest resistance, with PPr values of 0.75 and 0.74, respectively. Resistance to tetracyclines and aminopenicillins in the *E*. *coli* (PPr = 0.78 and 0.76, respectively) and *Salmonella spp.* (PPr = 0.72 and 0.73, respectively) subgroups was nearly the same. A high level of heterogeneity was observed among the studies listed in the tetracycline and aminopenicillins, with *I*² values of 87.5% and 86.3%, respectively (Figs. 2 and 3).

Monobactams (*n =* 6) and 3rd, 4th, and 5th generation cephalosporins (*n =* 19) had the lowest resistance, with PPr values of 0.30 and 0.34, respectively. The lowest heterogeneity was observed among the studies in the group, with an *I*² value of 76.0%, whereas for cephalosporins, *I*² was 92.1%. For the quinolone and fluoroquinolone groups (*n =* 23), colistin (*n =* 5), aminoglycosides (*n =* 20), and macrolides and ketolides (*n =* 6), the PPr values were 0.64, 0.55, 0.52, and 0.51, respectively.

#### Phenotypic resistance among non-Enterobacteriaceae

**Figure 4.**
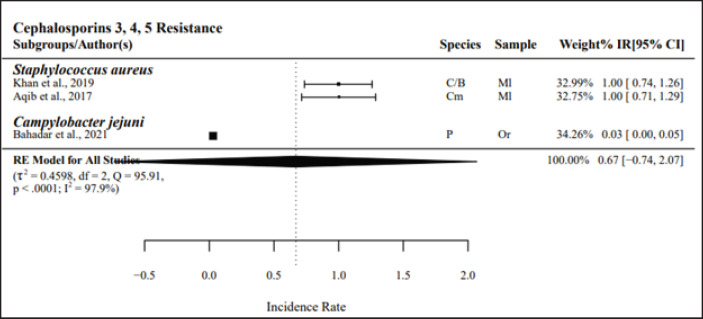
Pooled prevalence rates of antibiotic resistance for non-*Enterobacteriaceae.* IR, raw incidence rate; *I*^2^ = heterogeneity; RE, random effects model; Q, heterogeneity statistics.

**Figure 5.**
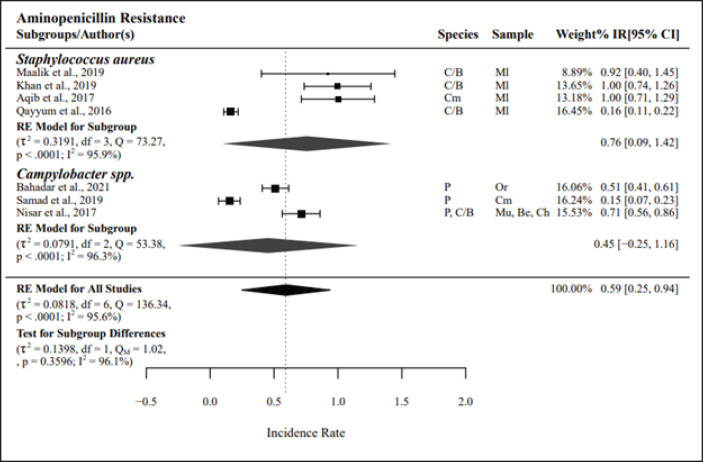
Pooled prevalence rates of antibiotic resistance for non-*Enterobacteriaceae.* IR, raw incidence rate; *I*^2^ = heterogeneity; RE, random effects model; Q, heterogeneity statistics.

The groups’ 3rd-, 4th-, and 5th-generation cephalosporins (*n =* 3) and aminopenicillins (*n* = 7) had the highest resistance, with PPr values of 0.67 and 0.59, respectively. Among aminopenicillins, the subgroup *Staphylococcus aureus* presented high resistance, with a PPr value of 0.76, whereas *Campylobacter* spp. had a PPr value of 0.45. The studies on the group’s 3rd-, 4th-, and 5th-generation cephalosporins and aminopenicillins presented high heterogeneity, with *I*² values of 97.9% and 95.6%, respectively (Figs. 4 and 5).

Aminoglycosides (*n =* 7) and quinolones and fluoroquinolones (*n =* 8) showed the lowest resistance among non-Enterobacteriaceae, with PPr values of 0.29 and 0.38, respectively. These groups also presented high study heterogeneity, with *I*² values of 89.8% and 92.3%, respectively. The PPr value for the tetracycline group (*n =* 6) was 0.45, whereas that for the macrolides and ketolides (*n =* 5) was 0.42. Phenotypic resistance to methicillin-resistant *Staphylococcus aureus* (MRSA) was studied in *n =* 4 studies, with a PPr value of 0.51 and *I*² of 90.5%.

Our analysis revealed that the funnel plots demonstrated a limited presence of outliers, with a notable exception. This anomaly can be attributed to the small sample size of the studies (*n =* 3) included in this specific forest plot, further compounded by the division into two distinct species.

## Discussion

Antimicrobials and their use have brought an extraordinary positive effect on the health and longevity of human life and led to a drastic reduction in mortality and morbidity associated with infectious diseases [22]. In contrast, this great success in human health was thwarted by the spread of antimicrobial-resistant strains that have continued to emerge and threaten to reverse the gains achieved over the last several decades [23]. AMR is now a global health issue. The globalization of the food system, with increased movement of livestock and agricultural products along with human travel, facilitates the rapid spread and mixing of AMR genes [24]. If proactive solutions are not implemented to curb the rise of drug resistance, it is projected that by 2050, approximately 10 million lives per year and a cumulative economic output of 100 trillion USD will be jeopardized owing to drug-resistant infections. Approximately 700,000 individuals succumb to antibiotic-resistant infections annually [25]. Worldwide, there is a huge imbalance between the demand and supply of antimicrobials because large quantities of antibiotics are wasted on patients and animals who do not need them, whereas those in need of them do not have access.

This systematic review and meta-analysis focus on assessing the extent of AMR in food animals in Pakistan. Using a one-health approach, the goal was to quantify how animals contribute to resistance in the country’s food production sector. The analysis covered all Global Antimicrobial Resistance and Use Surveillance System-reported microbes, including *E*. *coli*, *Klebsiella*, *Pseudomonas*, MRSA, and *Acinetobacter baumannii,* among food animals. As per our study, the high levels of antibiotic resistance in *E*. *coli* (38%) and *Salmonella* (31%) are concerning because these bacteria are common causes of foodborne illnesses and can spread between animals and humans. Similarly, the resistance found in *Staphylococci* (23%) and *Campylobacter* (19%) is significant, as these bacteria contribute to both human and animal infections, with *Campylobacter* being a major cause of gastrointestinal infections worldwide. Although *Klebsiella* had a lower resistance rate (18%), its role in hospital-acquired infections remains concerning. According to 2022 surveillance data on AMR in Europe, the reporting laboratories also most frequently documented *E*. *coli*, comprising 39.2% of reported cases. Moreover, more than half of *E*. *coli* and one-third of *Klebsiella* isolates reported by the European Antimicrobial Resistance Surveillance Network were resistant to at least one antibiotic [26]. Furthermore, the detection of MDR colistin-resistant *E*. *coli* strains with 100% resistance to colistin, gentamicin, florfenicol, tetracycline, cefotaxime, and trimethoprim, as reported in a study from China [27].

We have seen the high resistance of *E*. *coli* and *Salmonella* to ampicillin and ciprofloxacin, which is alarming as these antibiotics are commonly used to treat infections in humans. The significant resistance to chloramphenicol and tetracycline further underscores the persistence of older antibiotics in resistance selection. *Staphylococci*’*s* high resistance to cefoxitin and penicillin raises concerns about the prevalence of methicillin-resistant strains. The strikingly high ciprofloxacin resistance in *Campylobacter* (50.4%) and *Klebsiella* (83.3%) is particularly concerning, as fluoroquinolones are critical for treating severe infections. Similarly, according to the European Union summary report on AMR in zoonotic and indicator bacteria from humans, animals, and food during 2019–2020, *E*. *coli* and *Salmonella* from EU member countries were the most resistant to ampicillin, tetracyclines, and sulfonamides, whereas *Campylobacter jejuni* and *C*. *coli* were the most resistant to ciprofloxacin [28]. In 2014, New Zealand reported the highest resistance of *Campylobacter* to fluoroquinolones [29]. In China, the resistance to ciprofloxacin in *Campylobacter* isolated from broilers was exceptionally high at 99.2% between 2012 and 2014, 74.3% in Europe (eight countries) in 2014, and 69.7% in Italy in 2015 [30]. The high level of resistance in *Campylobacter* against ciprofloxacin, despite restrictions on its use in animals, indicates horizontal transfer of resistance among species. In relation to *Klebsiella* resistance, besides the elevated resistance to ciprofloxacin attributed to its use in animals [31], European countries experienced a significant increase in carbapenem resistance in *Klebsiella* from 2016 to 2020 [26]. Similarly, a meta-analysis of studies from Southeast Asia documented carbapenem-resistant *E*. *coli* and *Klebsiella* in 8 and 9 of 11 nations, respectively, with particularly high resistance rates (> 5%) in Indonesia, the Philippines, Thailand, and Vietnam [32].

According to our analysis, studies (2016–2020) consistently reported MDR in *E*. *coli* across all 12 studies, whereas *Salmonella*, *Staphylococci*, *Campylobacter*, and *Klebsiella* exhibited varying degrees of resistance in multiple studies. During 2015–2017, Haulisah et al. [33] found *E*. *coli* and *Staphylococcus aureus* isolated from ruminants in Malaysia to be 67.0% and 65.6% MDR. Between 2014 and 2019, in Portugal, there was the highest MDR prevalence (74%–90%) for *E*. *coli* from animals, followed by *Salmonella* (36.0%) and *Campylobacter* (17.0%) from poultry/poultry products. This study also reported that *Campylobacter* was the most resistant to ciprofloxacin in Portugal [34], similar to our findings.

The meta-analysis revealed significant resistance of Enterobacteriaceae to tetracyclines (PPr = 0.75) and aminopenicillins (PPr = 0.74), with *E. coli* and *Salmonella* spp. subgroups exhibiting similar levels of resistance. Considerable heterogeneity was noted in the studies, with I^2^ values of 87.5% for tetracyclines and 86.3% for aminopenicillin. However, in non-Enterobacteriaceae, cephalosporins and aminopenicillins showed the highest resistance, yielding PPr values of 0.67 and 0.59, respectively. Within the aminopenicillin subgroup, *Staphylococcus aureus* showed high resistance (PPr = 0.76), while *Campylobacter* spp. exhibited a PPr value of 0.45. Notably, the studies investigating cephalosporins and aminopenicillins revealed significant heterogeneity, reflected by I² values of 97.9% and 95.6%, respectively.

In food animals, the high incidence of AMR and MDR may be linked to poor farming practices, inadequate slaughterhouse protocols, insufficient hygiene measures, and the absence of policy and legislative frameworks on antimicrobial use (AMU) in Pakistan [35]. The high prevalence of AMR and MDR observed in these studies is particularly alarming because of the potential mobility of associated antibiotic-resistance genes in genetic elements. It is important to acknowledge that the included studies may not represent all regions of Pakistan equally, which may affect the generalizability of the findings. AMR and MDR in *E*. *coli* can lead to severe human infections and serve as reservoirs for antibiotic resistance and virulence genes. These genes can be transmitted to other bacteria, both commensal and pathogenic, and spread through the food chain. Therefore, monitoring AMR in indicator bacteria, such as *E*. *coli,* in food animals and related products is crucial. This monitoring is essential for understanding the evolution and transmission patterns of antibiotic-resistant bacteria as well as the dissemination of antibacterial resistance and virulent genes within the food chain.

Although this study did not specifically quantify antibiotic usage in food animals, the significant prevalence of AMR and MDR among bacterial isolates, such as *E*. *coli, Salmonella, Staphylococci, Campylobacter*, and *Klebsiella,* suggests widespread antibiotic use in Pakistani farming practices. A recent study conducted on the quantification of AMU in commercial poultry in Pakistan reported that overall 60% of the antibiotics used in broiler chickens were critically important antimicrobial classes for human medicine as characterized by the WHO, and the top three antibiotics used were neomycin (111.39 mg/PCU), doxycycline (91.91 mg/PCU), and tilmicosin (77.22 mg/PCU) in the summer season, while doxycycline (196.81 mg/PCU), neomycin (136.74 mg/PCU), and amoxicillin (115.04 mg/ PCU) were the top used in the winter [36].

Antibiotic-resistant and MDR bacteria have been extensively observed in food animals in farm and abattoir settings. The MDR bacteria identified in food animals on farms could be associated with antimicrobial usage practices in these settings. However, the presence of MDR bacteria at abattoirs raises food safety concerns by potentially exposing consumers.

The emergence and spread of antibiotic-resistant and MDR bacteria in Pakistan pose a significant public health threat. With the globalization of trade in food animals and products, as well as increased international travel, the spread of these bacteria transcends geographic boundaries. The findings emphasize the need to conduct more high-quality research. Establishing and implementing a minimum set of monitoring system criteria is crucial. Furthermore, collaboration among various sectors and disciplines must be strengthened, and guidance from the Quadripartite Joint Secretariat on AMR and the Quadripartite Technical Group on AMR/U Integrated Surveillance could be helpful.

It is important to consider certain limitations when interpreting this study, such as the restricted geographical representation (limited to major cities), the specific population targeted, the study design, and the absence of data on antibiotic utilization or consumption in humans and food animals, due to data scarcity. Owing to the scarcity of studies in this area, it was impossible to determine the resistance levels to specific antimicrobials, particularly those considered “critically important” in animal and human health, and their correlation with resistance genes and virulence factors. The inclusion criteria and subgroup analyses used in this study helped to reduce heterogeneity; however, it could not be confidently assumed that the studies were fully comparable.

As Pakistan has started to make progress on AMR monitoring and surveillance in food and agriculture sectors through the development of national AMR surveillance strategies for healthy food animals, diseased food animals, the food animal environment, and aquaculture, and national standard operating procedures for sample collection and shipment, bacteria isolation and characterization, laboratory biosecurity, AST, and data recording during 2020–2022 [37], it would be interesting to conduct another meta-analysis and systematic review capturing at least a 10-year period to gauge how the guidelines have helped the country in managing AMR and AMU in food animal production in Pakistan. In addition, it would be important to include aquaculture, as more and more attention is being paid to this sector, and guidelines are being developed for monitoring AMR in aquaculture [38]. Finally, the findings and recommendations from Qiu et al. [37] are crucial for Pakistan to revise and update its AMR NAP and appropriately reflect activities in the food and agricultural sectors. Despite raising awareness and building surveillance capacities, AMR initiatives such as the One Health AMR Strategy and Fleming Fund interventions reveal gaps in policy enforcement, antibiotic stewardship, data sharing, and sector coordination.

## Conclusion

AMR is a serious global health threat, and it is estimated that 1.27 million deaths in 2019 are directly attributable to bacterial AMR [39]. An analysis of studies from 2016 to 2020 highlights significant AMR concerns in food animals in Pakistan. The results revealed that *E*. *coli* in food animals showed the highest resistance to amikacin, piperacillin-tazobactam, ampicillin, cephalosporins, quinolones, tetracyclines, and chloramphenicol, with lower resistance to carbapenems and nitrofurantoin. *Klebsiella* spp. had high resistance to ciprofloxacin, flumequine, doxycycline, and chloramphenicol. *S*. *aureus* showed significant resistance to cefoxitin, penicillin, and oxytetracycline, with MRSA emerging in dairy animals. MDR MRSA in table eggs poses a food safety risk and could spread resistant strains to humans. MDR *Salmonella* Typhimurium, Enteritidis, and *Campylobacter jejuni*, prevalent in food animals, also pose major public health threats. The presence of virulent genes is a potential threat to resistance transmission. Given these findings, urgent and sustained measures are required, including the enforcement of strict control systems, adherence to food safety regulations, and restrictions on antibiotic use in animal farming, agriculture, and human medicine. Strengthening national AMR surveillance and antimicrobial stewardship programs is critical for mitigating the spread of resistant pathogens. Future policies should focus on minimizing antimicrobial misuse in livestock production, enhancing surveillance systems, and promoting alternative strategies such as vaccination and improved biosecurity practices.

## List of abbreviations

AMR, antimicrobial resistance

AMU, antimicrobial use

AST, antimicrobial susceptibility testing

CLSI, Clinical and Laboratory Standards Institute

CLSI, Clinical and Laboratory Standards Institute

FAO, Food and Agriculture Organization

GS, Google Scholar

MDR, Multidrug resistant

MRSA, methicillin-resistant *Staphylococcus aureus*

NAP, National Action Plan

NCCLS, National Committee for Clinical Laboratory Standards

NCCLS, National Committee for Clinical Laboratory Standards

WHO, World Health Organization

WOAH, World Organization for Animal Health

WoS, Web of Science

XDR, Extensive drug resistance.
